# Anlotinib Reverses Multidrug Resistance (MDR) in Osteosarcoma by Inhibiting P-Glycoprotein (PGP1) Function In Vitro and In Vivo

**DOI:** 10.3389/fphar.2021.798837

**Published:** 2022-01-17

**Authors:** Gangyang Wang, Lingling Cao, Yafei Jiang, Tao Zhang, Hongsheng Wang, Zhuoying Wang, Jing Xu, Min Mao, Yingqi Hua, Zhengdong Cai, Xiaojun Ma, Shuo Hu, Chenghao Zhou

**Affiliations:** ^1^ Department of Orthopaedics, Shanghai General Hospital, Shanghai Jiao Tong University School of Medicine, Shanghai, China; ^2^ Shanghai Bone Tumor Institute, Shanghai, China; ^3^ Department of Rehabilitation, Shanghai Fifth Rehabilitation Hospital, Shanghai, China

**Keywords:** anlotinib, osteosarcoma, multidrug resistance, ATP-binding cassette (ABC) transporter, P-glycoprotein

## Abstract

Overexpression of the multidrug resistance (MDR)-related protein P-glycoprotein (PGP1), which actively extrudes chemotherapeutic agents from cells and significantly decreases the efficacy of chemotherapy, is viewed as a major obstacle in osteosarcoma chemotherapy. Anlotinib, a novel tyrosine kinase inhibitor (TKI), has good anti-tumor effects in a variety of solid tumors. However, there are few studies on the mechanism of anlotinib reversing chemotherapy resistance in osteosarcoma. In this study, cellular assays were performed *in vitro* and *in vivo* to evaluate the MDR reversal effects of anlotinib on multidrug-resistant osteosarcoma cell lines. Drug efflux and intracellular drug accumulation were measured by flow cytometry. The vanadate-sensitive ATPase activity of PGP1 was measured in the presence of a range of anlotinib concentrations. The protein expression level of ABCB1 was detected by Western blotting and immunofluorescence analysis. Our results showed that anlotinib significantly increased the sensitivity of KHOSR2 and U2OSR2 cells (which overexpress PGP1) to chemotherapeutic agents *in vitro* and in a KHOSR2 xenograft nude mouse model *in vivo*. Mechanistically, anlotinib increases the intracellular accumulation of PGP1 substrates by inhibiting the efflux function of PGP1 in multidrug-resistant cell lines. Furthermore, anlotinib stimulated the ATPase activity of PGP1 but affected neither the protein expression level nor the localization of PGP1. In animal studies, anlotinib in combination with doxorubicin (DOX) significantly decreased the tumor growth rate and the tumor size in the KHOSR2 xenograft nude mouse model. Overall, our findings suggest that anlotinib may be useful for circumventing MDR to other conventional antineoplastic drugs.

## Introduction

Osteosarcoma is one of the most common and most aggressive primary malignant bone tumors in children and adolescents, and the incidence of osteosarcoma has a second peak in adults over the age of 65 years (Mirabello et al., 2009). The standard treatment for osteosarcoma currently relies on conservative surgery and neoadjuvant chemotherapy, which has improved the survival rate from less than 20% prior to the 1970s to 65–75% today (Jaffe, 2009; Chen et al., 2017). Unfortunately, the cure rate has not increased over the past 25–30 years. A three-drug combination regimen is routinely selected for systemic chemotherapy of osteosarcoma in clinical practice (Gill and Gorlick, 2021). Through nearly three decades of clinical trial testing, the multiple combinations of methotrexate, doxorubicin, epirubicin, ifosfamide and etoposide before and after definitive surgical resection of osteosarcoma has achieved consistent efficacy outcomes, with an overall event-free survival (EFS) rate of 12% at 4 months (Meyers et al., 2008; Ritter and Bielack, 2010; Gill and Gorlick, 2021). One of the fundamental obstacles to the successful treatment of osteosarcoma is the development of multidrug resistance (MDR), which causes tumor cells to become resistant to structurally and mechanistically distinct classes of chemotherapeutic agents (Vasiliou et al., 2009; Yang et al., 2017).

Several mechanisms have been shown to promote anticancer drug resistance in osteosarcoma. One important molecular basis for MDR is overexpression of plasma membrane P-glycoprotein (PGP1), which actively extrudes a variety of chemotherapeutic agents from cancer cells, thereby significantly decreasing the efficacy and increasing the side effects of chemotherapeutic drugs (Gillet et al., 2007). Previous studies have shown that PGP1 is overexpressed in various multidrug-resistant osteosarcoma cell lines and drug-resistant osteosarcoma tissues (Yang et al., 2014; Ye et al., 2016; Duan et al., 2017; Lu et al., 2017). Hence, developing new chemotherapeutic agents to inhibit the efflux function and/or downregulate the expression of PGP1 may make it possible to overcome MDR and chemotherapy resistance.

Most previous investigations have aimed to reverse and prevent MDR by targeting PGP1, but success has been limited due to unacceptable toxicity and problematic pharmacokinetic interactions (Liu, 2009; Xue et al., 2016). Tyrosine kinase inhibitors (TKIs) are a class of pharmaceutical drugs that inhibit tyrosine kinases (Boutayeb et al., 2012). The antitumor mechanism of TKIs is believed to inhibit functions of ATP for binding to the ATP site of the catalytic domain of several oncogenic tyrosine kinases (Azzariti et al., 2010). Several TKIs, including imatinib, nilotinib, gefitinib, apatinib, and afatinib, have been reported to significantly attenuate or reverse MDR mediated by ATP-binding cassette (ABC) transporters (Dai et al., 2008; Mi et al., 2010; Ma et al., 2014; Wang et al., 2014). Anlotinib, a novel antitumor drug, is a receptor TKI with multiple targets, notably vascular endothelial growth factor receptor type 2 (VEGFR2), VEGFR3, platelet-derived growth factor b (PDGFRβ), and stem cell factor receptor (c-Kit) (Zhong et al., 2018a; Lin et al., 2018). The antitumor effect of anlotinib has been reported in many preclinical and clinical trials (Sun et al., 2016; Han et al., 2018a; Han et al., 2018b; Li, 2021). Because it acts at the ATP-binding site of the tyrosine kinase domains of VEGFR, anlotinib may inhibit the functions of ABC transporters by binding to their ATP-binding sites; this mechanism is similar to the mechanism by which several of the TKIs mentioned above operate to reverse MDR. In the current study, we determined that anlotinib significantly reverses PGP1-mediated MDR in human osteosarcoma cells *in vitro* and *in vivo*. Anlotinib, in combination with conventional antineoplastic drugs such as doxorubicin, could be a novel and effective therapy for the treatment of osteosarcoma patients.

## Materials and Methods

### Reagents

Anlotinib was obtained from Chia Tai Tianqing Pharmaceutical Group Co., Ltd. (Nanjing, China). All cell culture reagents were purchased from Invitrogen Life Technologies (Carlsbad, CA, United States). Antibodies specific for P-gp/ABCB1 and GAPDH were purchased from Cell Signaling Technology, Inc. (Danvers, MA, United States). Dimethyl sulfoxide (DMSO), DOX, paclitaxel, vincristine, rhodamine 123 (Rho-123), and other chemicals were purchased from Sigma-Aldrich (St. Louis, MO, United States).

### Cell Lines and Cell Culture

The multidrug-resistant human osteosarcoma cell lines KHOSR2 and U2OSR2 (established by DOX selection) and their respective drug-sensitive parental cell lines, KHOS and U2OS, were kindly provided by Dr. Zhenfeng Duan (University of California, Los Angeles, UCLA, CA, United States). These multidrug-resistant osteosarcoma cell lines have been extensively characterized in previous studies as having a stable MDR phenotype (Ye et al., 2016; Duan et al., 2017). Compared to the drug-sensitive cells (KHOS and U2OS), the drug-resistant cells (KHOSR2 and U2OSR2) overexpressed PGP1 (also known as ABCB1); furthermore, the expression of MRP1 (also known as ABCC1) and BCRP (also known as ABCG2) was undetectable in the drug-resistant cell lines (Duan et al., 2009a; Duan et al., 2009b). All cell lines were cultured in DMEM containing 10% FBS and 1% penicillin/streptomycin at 37°C in 5% CO_2_. All drug-resistant cell lines were periodically cultured with the affected drug to maintain their drug resistance characteristics. All cells were grown in drug-free culture medium for >2 weeks before being used for assays.

### Cell Cytotoxicity Assay

A cell counting kit-8 (CCK8, Dojindo, Kumamoto, Japan) assay was used to assess cell sensitivity to chemotherapeutic drugs, as described previously (Wang et al., 2017). Briefly, cell suspensions (3×10^4^/ml) were seeded into 96-well plates, incubated overnight and treated with increasing concentrations of anlotinib alone, chemotherapy drugs alone or a combination of both types of drugs. After incubation for 24 or 48 h, the cells were washed twice with PBS and incubated with CCK8 working solution for 2 h at 37°C according to the manufacturer’s protocol. The absorption was measured at 490 nm by an iMark microplate reader (Molecular Devices, Sunnyvale, United States). The IC50 values were calculated using a probit model (Li et al., 2015). The degree of resistance was estimated by dividing the IC50 of the multidrug-resistant cells by that of the drug-sensitive parental cells. The fold reversal factor for MDR was calculated by dividing the IC50 of cells treated with antitumor drugs in the absence of anlotinib by that of cells treated in the presence of anlotinib.

### Drug Accumulation Assay

To determine the effect of anlotinib on the intracellular accumulation of antitumor drugs, a flow cytometric assay was performed. Cells were seeded in six-well plates at a density of 5×10^5^/ml and treated with the indicated concentrations of anlotinib for 24 h. The cells were then incubated with 3 μM Rho-123 and 50 μM DOX for 2 h at 37°C. After incubation, the cells were harvested, washed 3 times with cold PBS, and analyzed by an Accuri C6 flow cytometer (BD Biosciences, Mountain View, CA, United States).

Fluorescence microscopy was used to visualize the effects of anlotinib on the intracellular accumulation of Rho-123 and DOX. Briefly, cells were seeded in six-well plates and exposed to Rho-123 and DOX with or without anlotinib pretreatment. The cells were washed twice with PBS, fixed with 4% paraformaldehyde for 20 min, permeabilized with 0.1% Triton X-100, and incubated with DAPI for 15 min. Fluorescence images were acquired by using a DMI3000B fluorescence microscope (Leica, Germany) and processed with LAS V4.3 software.

### Rho-123 Efflux Assay

A Rho-123 efflux assay was performed as described previously (Guo et al., 2018). Cells were seeded in six-well plates at a density of 5×10^5^/ml and treated with 3 μM Rho-123 for 30 min. The cells were then collected, washed three times with cold PBS and subsequently incubated with or without 0.4 μM anlotinib for 0, 30, 60, 90 or 120 min. The cells were then harvested at the designed time points, washed 3 times with cold PBS, and analyzed by an Accuri C6 flow cytometer.

### PGP1 ATPase Assay

The vanadate-sensitive ATPase activity of PGP1 was measured as previously described (Ambudkar, 1998). Briefly, crude membranes isolated from High Five insect cells expressing PGP1 were incubated with various concentrations of anlotinib for 5 min. The ATPase reaction was then initiated by the addition of 5 mmol/l Mg-ATP to a 100 μl total reaction mixture. After 20 min of incubation at 37°C, 10% SDS solution was added to terminate the reaction. The amount of inorganic phosphate (IP) released was detected at 880 nm by a microplate spectrophotometer.

### Western Blot Analysis

Cells were seeded at a density of 500 cells/well in six-well plates evenly. After 24 h, cells were treated with various concentration of anlotinib (0, 0.1, 0.2, and 0.4 μM) for about 24 h. Cell samples were lysed for 30 min in ice-cold radioimmunoprecipitation assay (RIPA) buffer containing a protease inhibitor cocktail (Sigma-Aldrich). The cell lysates were centrifuged at 12,000 g for 15 min at 4°C, and the supernatant was collected. Then, the protein concentrations were quantified using a BCA Protein Assay (Thermo Scientific, Fremont, CA, United States). Equivalent amounts of protein were loaded and separated by SDS-PAGE, and the proteins were then transferred to polyvinylidene difluoride (PVDF) membranes (Millipore, Billerica, MA, United States). After blocking with 5% nonfat milk in PBST buffer for 1 h at room temperature, the membranes were incubated with primary antibodies at 4°C overnight. The membranes were washed with TBST and then incubated with secondary antibodies (Sigma-Aldrich, Inc.) for 1 h at room temperature. Bands were detected by an enhanced chemiluminescence kit (Millipore, Billerica, MA, United States).

### Immunofluorescence Analysis

Cells were seeded at a density of 500 cells/well on coverslips. After 24 h, cells were treated with various concentration of anlotinib (0, 0.1, 0.2, and 0.4 μM) for 24 h. After incubation, the cells were fixed with 4% paraformaldehyde, permeabilized with 0.1% Triton X-100 and blocked for 1 h with 6% BSA. Then, the cells were incubated with the indicated primary antibodies at 4°C overnight, followed by incubation with an Alexa Fluor 555-conjugated secondary antibody (1:1,000) for 1 h. Nuclei were stained with DAPI solution. Images were acquired with a confocal microscope (Leica, Wetzlar, Germany) and analyzed by image J software.

### Animal Experiments

All animal care and experimentation procedures were conducted according to the relevant guidelines with the approval of the Institutional Animal Care and Use Committee of the Shanghai Jiao Tong University School of Medicine. Four-week-old male BALB/c-nu mice (Shanghai SLAC Laboratory Animal Co., Ltd., Shanghai, China) weighing approximately 20 g were purchased from the Shanghai Laboratory Animal Center of the Chinese Academy of Sciences and provided with sterilized food and water in a standard animal laboratory.

A multidrug-resistant osteosarcoma orthotopic xenograft model was established as previously described, with minor modifications (Wang et al., 2017). Briefly, 1×10^6^ KHOSR2 cells were suspended in 20 μl of sterile PBS and implanted into the tibial medullary cavity of each mouse. One week after cell inoculation, the mice were randomized into four groups (n = 5 per group) and received various treatments: 1) saline every other day (qod); 2) DOX (3 mg/kg, intraperitoneal (ip) injection, qod); 3) anlotinib (2 mg/kg, intragastric administration, qod); and 4) anlotinib (2 mg/kg, intragastric administration, qod, given 1 h before DOX administration) plus DOX (3 mg/kg, ip, qod). Mouse body weights and tumor sizes were measured every 2 days to observe dynamic changes in tumor growth. Tumor volumes were calculated by a standard formula: length × width^2^/2. After 18 days of treatment, all mice were euthanized, and the tumors were harvested and weighed.

### Statistical Analysis

Data are presented as the mean ± SD. All experiments were performed at least three times, and differences were determined by using Student’s t-test or One-way ANOVA. *p*-values < 0.05 (*) were considered statistically significant.

## Results

### Anlotinib Reverses MDR in Osteosarcoma Cells

**FIGURE 1:**
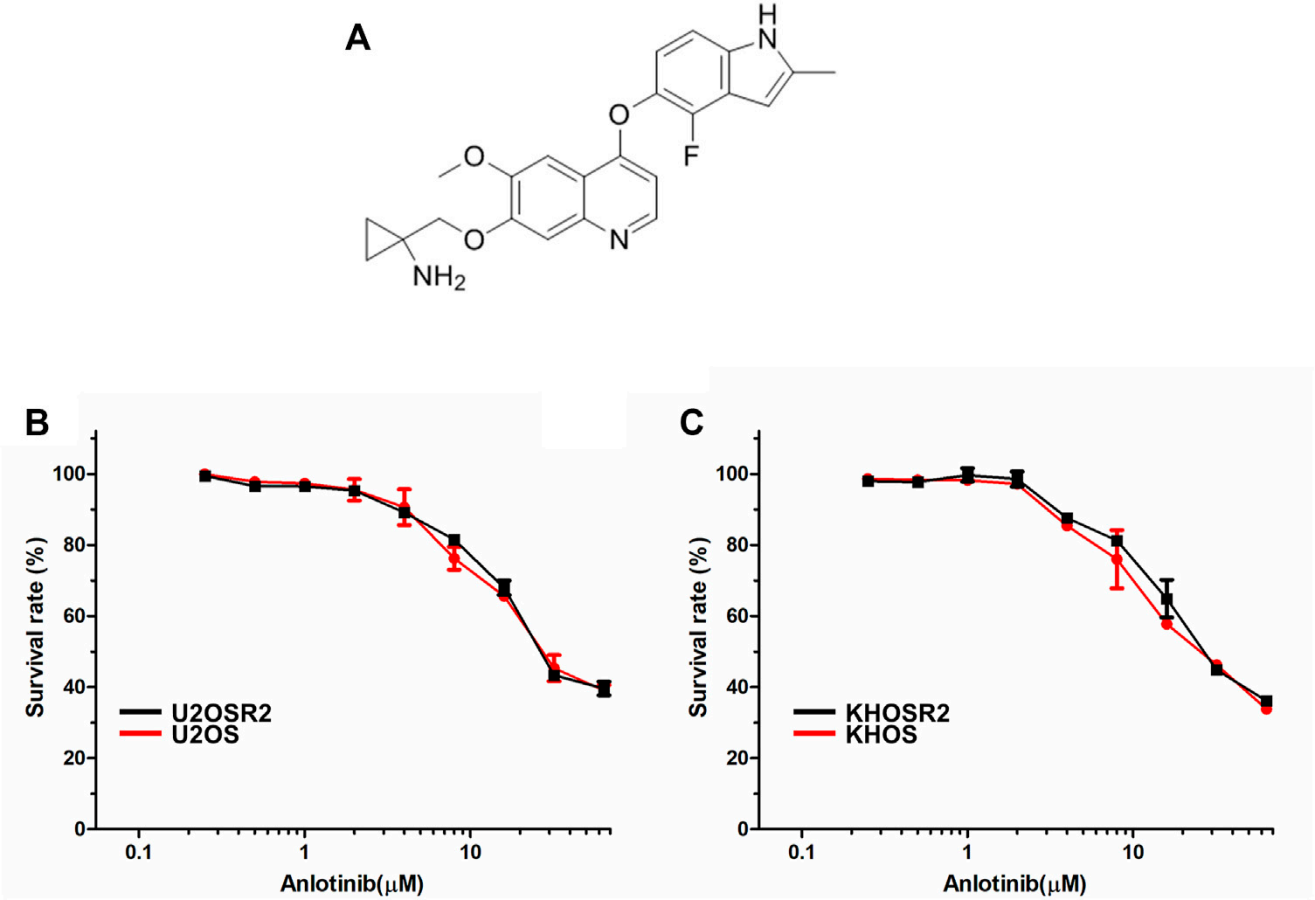
The structure of anlotinib and the cytotoxicity of anlotinib in drug-resistant osteosarcoma cell lines and their drug-sensitive parental cell lines. **(A)**. The structure of anlotinib. Osteosarcoma drug-resistant and their drug-sensitive cell lines were treated with anlotinib at the indicated concentrations for 48 h. Cell viability was measured by CCK8 **(B)**. Cytotoxicity curves for the U2OSR2 and U2OS cell lines incubated with anlotinib alone. **(C)**. Cytotoxicity curves for the KHOSR2 and KHOS cell lines incubated with anlotinib alone. The data were shown as the mean ± SD from three independent experiments.

The structure of anlotinib is shown in Figure 1A. The cytotoxicity of anlotinib in different cell lines was analyzed by a CCK8 assay. The IC50 values were 32.07, 32.41, 27.17, and 30.64 μM for U2OS, U2OSR2, KHOS, and KHOSR2 cells, respectively (Figure 1B). Based on the cytotoxicity curves, we selected 0.4 μM as the maximum anlotinib concentration; at this concentration, the cell viability in all cell lines used in the MDR reversal study was greater than 90%.

**FIGURE 2:**
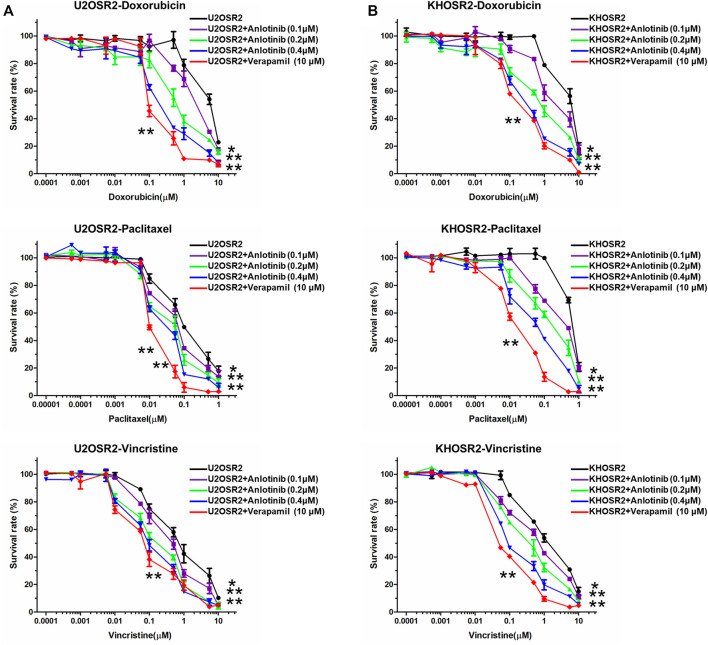
Effect of anlotinib on the reversal of drug resistance in multidrug-resistant osteosarcoma cell lines. Cells were treated with chemotherapeutic drugs and anlotinib at the indicated concentrations. The relative sensitivity of each line to chemotherapeutic drugs was determined by a CCK8 assay 48 h after treatment. **(A)**. Reversal of drug resistance by anlotinib in U2OSR2 cells. **(B)**. Reversal of drug resistance by anlotinib in KHOSR2 cells. Data represent the mean ± SD of at least three independent experiments (*p < 0.05; **p < 0.01).

To determine whether anlotinib can reverse MDR in osteosarcoma, cell survival assays were performed in the presence or absence of anlotinib. The IC50 values of antineoplastic drugs in sensitive and resistant cells treated with different concentrations of anlotinib are shown in Table 1. As shown in Table 1 and Figure 2, compared to its effect in the U2OS and KHOS cell lines, anlotinib significantly potentiated the cytotoxicity of DOX, paclitaxel and vincristine in the U2OSR2 and KHOSR2 cell lines in a concentration-dependent manner. However, in the parental cell lines KHOS and U2OS, which do not express PGP1, anlotinib did not modulate the activity of these cytotoxic agents (Table 1). Verapamil at a concentration of 10 μM was used as a positive control PGP1 inhibitor. The above results suggest that anlotinib significantly sensitizes multidrug-resistant osteosarcoma cells to antineoplastic drugs that are substrates of PGP1.

**TABLE 1:** Ability of anlotinib to reverse drug resistance in multidrug-resistant osteosarcoma cell lines.

Effect of anlotinib on reversing PGP1-mediated MDR in osteosarcoma cells				
Compounds	IC50 ± SD (μM; fold-reversal)			
	U2OS		U2OSR2	
Doxorubicin	0.1648 ± 0.0050	(1.00)	4.694 ± 0.1368	(1.00)
plus anlotinib 0.1 μM	0.1634 ± 0.0084	(1.01)	2.095 ± 0.0862	(2.24)
plus anlotinib 0.2 μM	0.1602 ± 0.0047	(1.03)	0.6648 ± 0.0519	(7.06)
plus anlotinib 0.4 μM	0.1683 ± 0.0088	(0.98)	0.2583 ± 0.0582	(18.17)
plus verapamil 10 μM	0.1509 ± 0.0067	(1.09)	0.1563 ± 0.0622	(30.03)
Paclitaxel	0.0140 ± 0.0019	(1.00)	0.1220 ± 0.0524	(1.00)
plus anlotinib 0.1 μM	0.0151 ± 0.0008	(0.93)	0.0646 ± 0.0188	(1.89)
plus anlotinib 0.2 μM	0.0128 ± 0.0016	(1.09)	0.0407 ± 0.0083	(2.99)
plus anlotinib 0.4 μM	0.0123 ± 0.0009	(1.13)	0.0298 ± 0.0052	(4.09)
plus verapamil 10 μM	0.0092 ± 0.0071	(1.52)	0.0139 ± 0.0051	(8.78)
Vincristine	0.0815 ± 0.0036	(1.00)	0.7484 ± 0.0501	(1.00)
plus anlotinib 0.1 μM	0.0782 ± 0.0088	(1.04)	0.3327 ± 0.0476	(2.24)
plus anlotinib 0.2 μM	0.0693 ± 0.0093	(1.18)	0.1716 ± 0.0499	(4.36)
plus anlotinib 0.4 μM	0.0639 ± 0.0087	(1.28)	0.1169 ± 0.0515	(6.40)
plus verapamil 10 μM	0.0686 ± 0.0063	(1.18)	0.0843 ± 0.0143	(8.87)
	KHOS		KHOSR2	
Doxorubicin	0.2496 ± 0.0690	(1.00)	4.795 ± 0.1891	(1.00)
plus anlotinib 0.1 μM	0.2170 ± 0.0751	(1.15)	2.185 ± 0.0828	(2.19)
plus anlotinib 0.2 μM	0.2372 ± 0.0604	(1.05)	0.7595 ± 0.0527	(6.31)
plus anlotinib 0.4 μM	0.2217 ± 0.012	(1.13)	0.3148 ± 0.0430	(15.23)
plus verapamil 10 μM	0.2322 ± 0.0431	(1.07)	0.2204 ± 0.0412	(21.76)
Paclitaxel	0.0213 ± 0.0067	(1.00)	0.6503 ± 0.0539	(1.00)
plus anlotinib 0.1 μM	0.0193 ± 0.0071	(1.10)	0.3071 ± 0.0693	(2.12)
plus anlotinib 0.2 μM	0.0175 ± 0.0045	(1.21)	0.1500 ± 0.0094	(4.34)
plus anlotinib 0.4 μM	0.0182 ± 0.0064	(1.17)	0.0583 ± 0.0082	(11.15)
plus verapamil 10 μM	0.01594 ± 0.0038	(1.33)	0.0174 ± 0.0035	(37.37)
Vincristine	0.0748 ± 0.0069	(1.00)	1.3440 ± 0.0498	(1.00)
plus anlotinib 0.1 μM	0.0683 ± 0.0084	(1.09)	0.6739 ± 0.0387	(1.99)
plus anlotinib 0.2 μM	0.0663 ± 0.0071	(1.29)	0.3608 ± 0.0483	(3.72)
plus anlotinib 0.4 μM	0.0680 ± 0.0085	(1.10)	0.1466 ± 0.0091	(9.16)
plus verapamil 10 μM	0.0675 ± 0.0038	(1.11)	0.0684 ± 0.0043	(19.65)

### Anlotinib Increased the Intracellular Accumulation of DOX and Rho-123 in Multidrug-Resistant Osteosarcoma Cells

**FIGURE 3:**
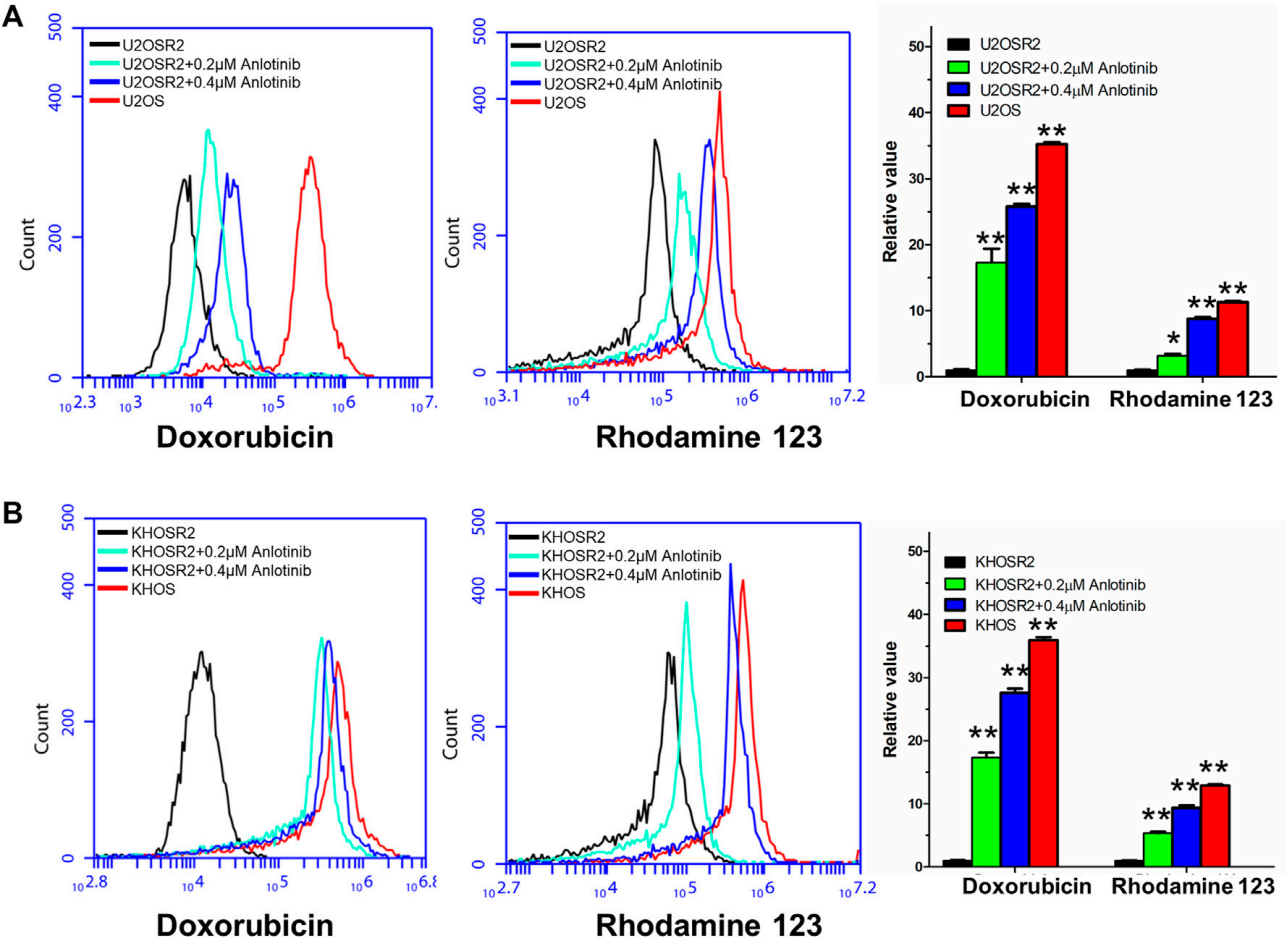
Effect of anlotinib on the intracellular accumulation of DOX and Rho-123 in multidrug-resistant osteosarcoma cells and their respective drug-sensitive parental cells. **(A)**. The accumulation of DOX and Rho-123 in U2OSR2 and U2OS cells was measured by flow cytometric analysis. **(B)**. The accumulation of DOX and Rho-123 in KHOSR2 and KHOS cells was measured by flow cytometric analysis. The results were quantified as the fold change in fluorescence intensity between the drug-sensitive parental cells and the paired multidrug-resistant cells. In the presence of anlotinib, the intracellular accumulation of DOX and Rho-123 in the drug-resistant U2OSR2 and KHOSR2 cells significantly increased in a dose-dependent manner. Data represent the mean ± SD of at least three independent experiments (*p < 0.05; **p < 0.01).

**FIGURE 4:**
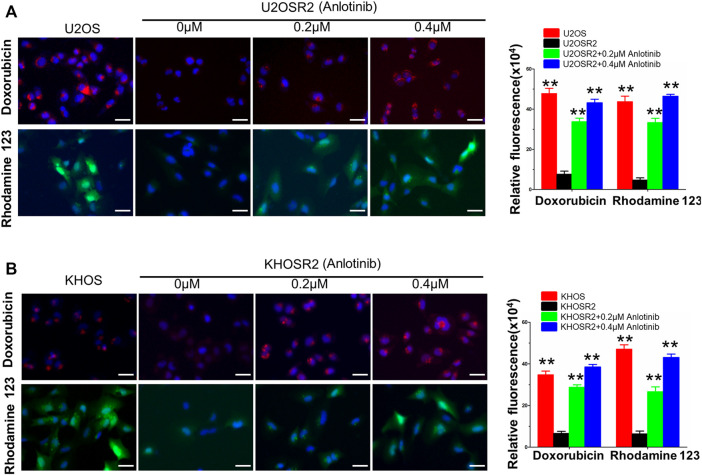
Anlotinib increases the intracellular accumulation of DOX and Rho-123 in multidrug-resistant osteosarcoma cells. To visualize the effects of anlotinib on the intracellular retention of DOX and Rho-123, multidrug-resistant osteosarcoma cells and their respective drug-sensitive parental cells were seeded in six-well plates and exposed to Rho-123 and DOX with/without anlotinib pretreatment. The cells were fixed and stained with DAPI. Fluorescence images were acquired by using a DMI3000B fluorescence microscope. **(A)**. Anlotinib increases the intracellular accumulation of DOX and Rho-123 in U2OSR2 and U2OS cells. **(B)**. Anlotinib increases the intracellular accumulation of DOX and Rho-123 in KHOSR2 and KHOS cells. Scale bars = 50 μm. The fluorescence integrated density was quantified and is represented by the lower lane bar graph. Data represent the mean ± SD of at least three independent experiments (*p < 0.05; **p < 0.01).

The above results indicated that anlotinib could significantly enhance the sensitivity of multidrug-resistant osteosarcoma cells to antineoplastic drugs. The mechanism by which this effect occurs is unknown. Therefore, to gain insight into the mechanism of action of anlotinib, we determined the intracellular accumulation of DOX and Rho-123 in multidrug-resistant osteosarcoma cells in the presence or absence of anlotinib by flow cytometric analysis and fluorescence imaging. As shown in Figures 3, 4, the intracellular accumulation of DOX and Rho-123 in the drug-resistant U2OSR2 and KHOSR2 cells was markedly lower than that in their respective drug-sensitive parental cells, U2OS and KHOS cells. In the presence of anlotinib, the intracellular accumulation of DOX and Rho-123 in the drug-resistant U2OSR2 and KHOSR2 cells significantly increased in a dose-dependent manner. This observation is consistent with the chemotherapy-sensitizing effect of anlotinib. These results suggested that anlotinib can increase the intracellular accumulation of chemotherapeutic agents in multidrug-resistant osteosarcoma cells.

### Anlotinib Decreased the Efflux of Rho-123 in Multidrug-Resistant Osteosarcoma Cells

**FIGURE 5:**
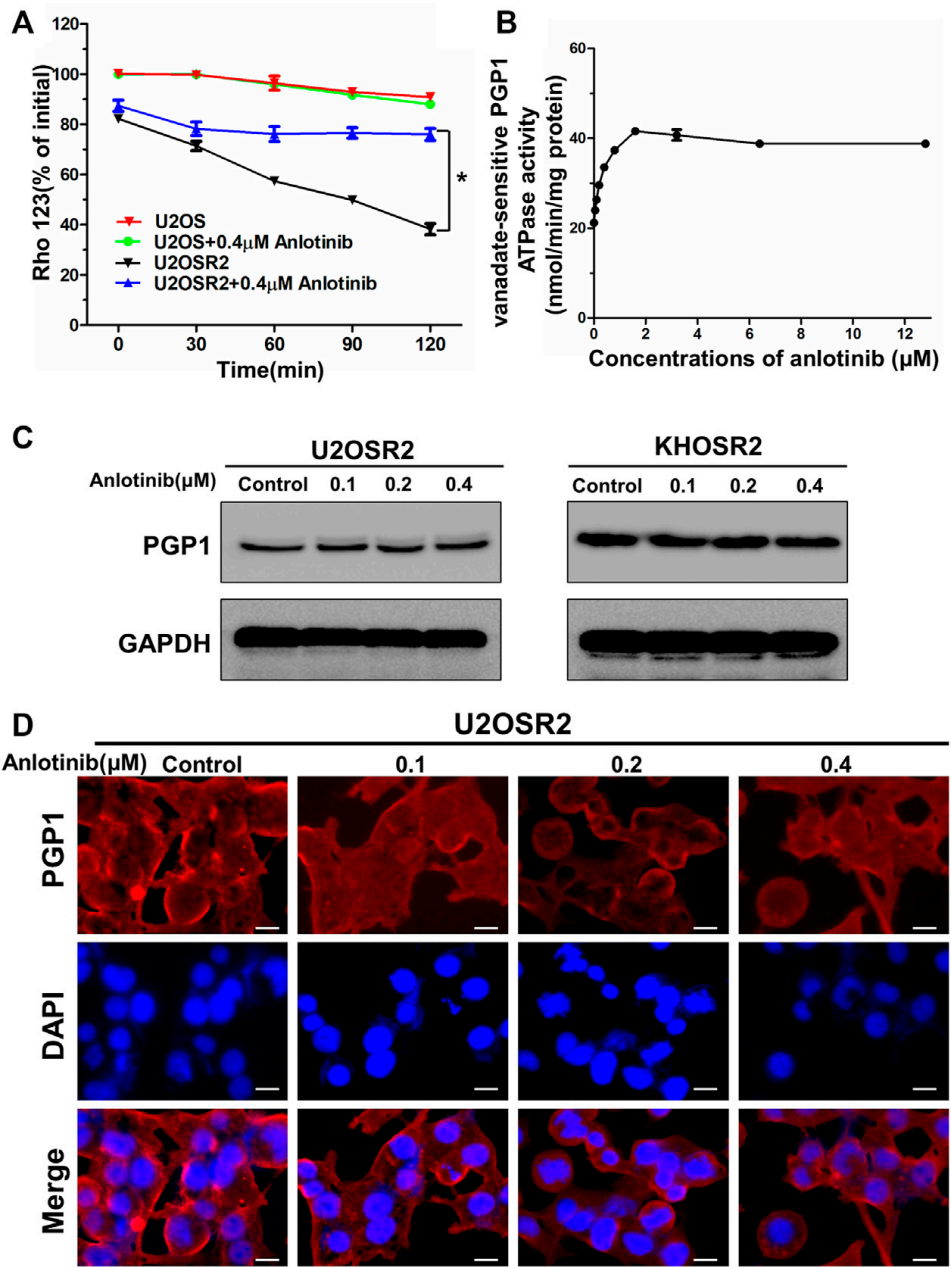
Effect of anlotinib on the efflux of Rho-123 and on the ATPase activity and expression level of PGP1 in multidrug-resistant osteosarcoma cells. **(A)**. The time course of Rho-123 efflux from multidrug-resistant osteosarcoma cells was measured in the presence or absence of 0.4 μM anlotinib. Data represent the mean ± SD of at least three independent experiments. *p < 0.05; **p < 0.01. **(B)**. Vanadate-sensitive PGP1 ATPase activity was evaluated in the presence of the indicated concentrations of anlotinib. **(C)**. Effect of 24 h of treatment with the indicated concentrations of anlotinib on the expression level of PGP1 in multidrug-resistant osteosarcoma cells. **(D)**. Effect of 24 h of treatment with the indicated concentrations of anlotinib on the subcellular localization of PGP1 in U2OSR2 cells. Scale bars = 20 μm.

To confirm whether the intracellular accumulation of DOX and Rho-123 was due to the inhibition of substrate drug efflux, we performed drug efflux assays in multidrug-resistant osteosarcoma cells in the presence or absence of anlotinib. We found that the efflux of Rho-123 from PGP1-overexpressing U2OSR2 cells was significantly higher than that from their drug-sensitive parental U2OS cells (Figure 5A). Treatment with anlotinib significantly decreased the efflux of Rho-123 from U2OSR2 cells, but it did not significantly alter the intracellular levels of Rho-123 in the parental cells. These results suggested that anlotinib increased the intracellular retention of Rho-123 by inhibiting PGP1-mediated efflux activity and that this effect was specific to PGP1-overexpressing U2OSR2 cells.

### Anlotinib Stimulated PGP1 ATPase Activity and did Not Affect PGP1 Expression

ABC transporters play a crucial role in the development of MDR by extruding drugs from cells. This process is coupled to the energy associated with ATP hydrolysis by the ATPase activity of ABC transporters, which is stimulated in the presence of transport substrates. To assess the effect of anlotinib on ATPase activity, we measured the vanadate-sensitive ATPase activity of PGP1 in the presence of a range of anlotinib concentrations. As shown in Figure 5B, anlotinib concentrations >2 μM stimulated the ATPase activity of PGP1 in a concentration-dependent manner, and this activity plateaued at approximately 42 nmol/min/mg protein and subsequently remained stable. Next, we further evaluated the effect of anlotinib on the expression level of PGP1 by Western blot and immunofluorescence analyses. Western blot analysis indicated that anlotinib did not directly interfere with the expression of PGP1 (Figure 5C), and the immunofluorescence assay indicated that anlotinib did not significantly alter the subcellular distribution of PGP1 in multidrug-resistant osteosarcoma cells. Therefore, the above data suggest that anlotinib may interact specifically with the PGP1 ATPase domain, which leads to inhibition of the efflux pump function of PGP1. In addition, treatment with anlotinib did not affect PGP1 expression.

### Anlotinib Potentiated the Anticancer Efficacy of DOX in a Multidrug-Resistant Osteosarcoma Cell Xenograft Model

**FIGURE 6:**
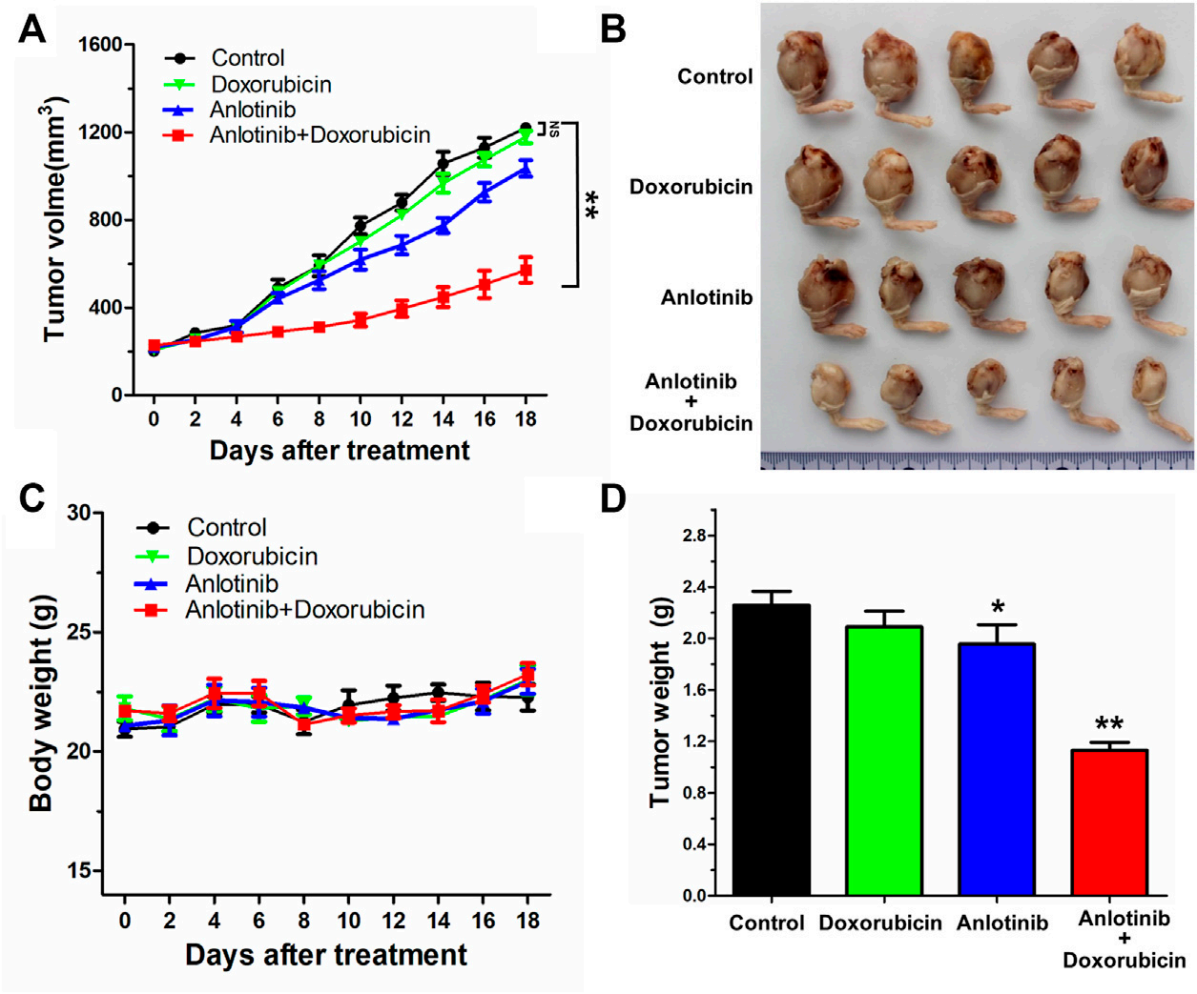
Potentiation of the antitumor effects of DOX by anlotinib in a KHOSR2 xenograft model in athymic nude mice. **(A)**. Changes in tumor volume over time in the KHOSR2 xenograft model are shown (n = 5). The data shown are the mean ± SD of the tumor volumes for each group. **(B)**. A representative image of the sizes of KHOSR2 tumors excised from different mice on the 18th day after implantation is shown. **(C)**. The body weights of the mice were measured every 2 days, and the average percent change after treatment was calculated. **(D)**. Mean tumor weights for the groups after tumor excision on the 18th day after implantation. The results are presented as the mean ± SD. *p < 0.05; **p < 0.01.

To explore whether anlotinib could reverse PGP1-mediated MDR *in vivo,* a previously established KHOSR2 xenograft model in nude mice was used. As shown in Figure 6A, there was no significant difference in tumor size between animals treated with saline and animals treated with DOX alone. However, the KHOSR2 tumor growth rate recorded over a period of 18 days was significantly lower in the anlotinib-DOX combination group than in the groups treated with saline, anlotinib alone or DOX alone (Figures 6A,B). Notably, treatment with 2 mg/kg anlotinib also slightly decreased the growth rate of KHOSR2 xenografts. Furthermore, at the doses tested, no mortality or apparent decrease in body weight was observed in the anlotinib-DOX combination group (Figure 6C). Taken together, these results indicate that anlotinib improved the efficacy of DOX in the KHOSR2 osteosarcoma xenograft model and did not increase the incidence of toxic side effects.

## Discussion

MDR is a major obstacle to the successful and effective chemotherapeutic treatment of cancer (Cancer multidrug resistan, 2000). In cancer cells, MDR produces resistance to numerous antineoplastic drugs that are structurally and mechanistically unrelated, and this resistance significantly decreases the efficacy of cancer chemotherapy (Krishna and Mayer, 2000). Numerous mechanisms, including the response to DNA damage, avoidance of apoptosis, induction of autophagy, overexpression of energy-dependent efflux proteins, activation of cancer stem cells, enhancement of drug efflux, and modification of cell cycle checkpoints, have been reported to mediate MDR (Gillet and Gottesman, 2010; Kartal-Yandim et al., 2016). One of the most important causes of MDR is the overexpression of ABC transporters, through which a wide range of structurally and functionally diverse antineoplastic drugs are extruded from tumor cells, thereby decreasing their intracellular accumulation and resulting in chemotherapeutic drug resistance (Pan et al., 2016). Previous studies have indicated that PGP1 is overexpressed in several multidrug-resistant osteosarcoma cell lines and drug-resistant osteosarcoma tissues (Rosier et al., 1995; Huang et al., 2012; Avnet et al., 2016). Therefore, inhibition of the drug transport function of PGP1 is a promising novel anticancer therapeutic strategy for reversing MDR in osteosarcoma. Most previous investigations have focused on reversing MDR by antagonizing the function or downregulating the expression of PGP1 in osteosarcoma, and several drugs to reverse MDR have been developed, including verapamil, cyclosporin A, biricodar and valspodar (Ferry et al., 1996; Mullin et al., 2004; Karthikeyan and Hoti, 2015). However, none of these drugs have been approved for clinical use in the reversal of MDR due to their detrimental toxic effects at the concentrations required to inhibit PGP1. In recent years, studies have demonstrated that several TKIs, including imatinib, nilotinib, gefitinib, apatinib, and afatinib, can inhibit the function of ABC transporters; the discovery of these TKIs represents a new strategy for reversing MDR. Anlotinib, a novel TKI, exhibits potent anticancer activity in many cancers, including renal cancer, non-small cell lung cancer, and sarcoma (Zhong et al., 2018b; Chi et al., 2018; Lin et al., 2018). More importantly, anlotinib suppresses the activity of VEGFR-2, PDGFRα/β, c-Kit, Ret, Aurora-B, colony stimulating factor 1 receptor (c-FMS), and discoidin domain receptor 1 (DDR1) (Han et al., 2018a; Chi et al., 2018; Xie et al., 2018). Based on these observations, we hypothesized that anlotinib might interact with ABC transporters and effectively overcome MDR.

**FIGURE 7:**
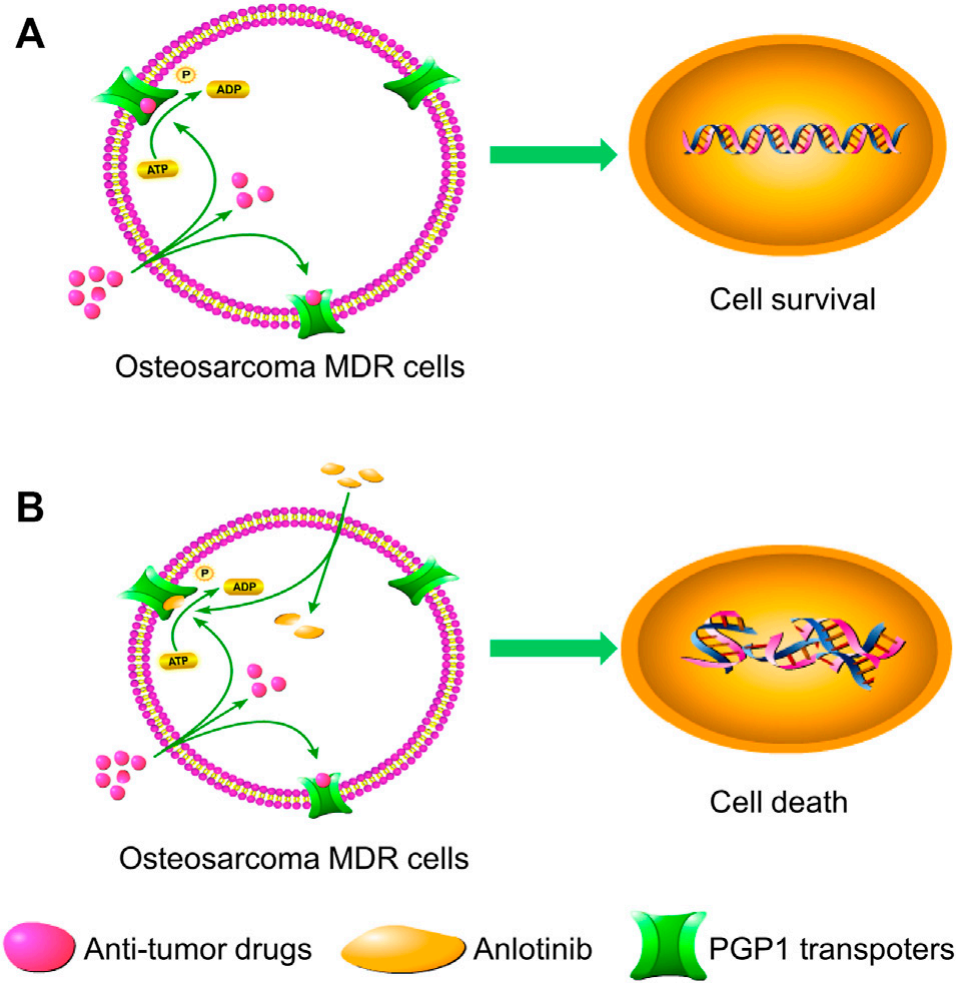
Schematic diagram showing the MDR reversal effect of anlotinib. **(A)**. In the absence of anlotinib, PGP1 transporters utilize energy derived from ATP hydrolysis to extrude their substrate drugs across the membrane. **(B)**. However, when anlotinib is present, it may bind to the ATP-binding site of PGP1, thereby blocking the efflux of substrate drugs through the transporter and increasing the intracellular accumulation of substrate drugs. Therefore, anlotinib can increase the intracellular concentration of substrate drugs in multidrug-resistant cells.

In the current study, we examined the effect of anlotinib on PGP1-mediated drug resistance *in vitro* and *in vivo*. In our cell viability assay, we used DOX-selected U2OSR2 and KHOSR2 osteosarcoma cells that have been extensively characterized as having both a stable MDR phenotype and PGP1 overexpression, consistent with previous reports (Ye et al., 2016). The cytotoxic effect of anlotinib in multidrug-resistant osteosarcoma cells and in the corresponding parental cells was not significantly different. Furthermore, treatment with 0.4 μM anlotinib (a concentration at which the cell viability was greater than 90%) significantly potentiated the efficacy of chemotherapeutic agents that are known PGP1 substrates in the multidrug-resistant U2OSR2 and KHOSR2 osteosarcoma cell lines. Moreover, anlotinib did not alter the sensitivity of the drug-sensitive parental U2OS and KHOS cells to chemotherapeutic agents. These results confirmed the chemotherapy-sensitizing effect of anlotinib in multidrug-resistant osteosarcoma cell lines. To further investigate whether anlotinib could enhance the efficacy of chemotherapeutic agents *in vivo*, we adopted a KHOSR2 xenograft nude mouse model. Anlotinib was found to significantly enhance the antitumor activity of the PGP1 substrate drug DOX in KHOSR2 cell xenografts without increasing its toxicity. Thus, our *in vitro* and *in vivo* results suggest that anlotinib may be a strong PGP1 inhibitor candidate, which supports further investigation of combination chemotherapy that includes anlotinib plus conventional anticancer drugs in cancer patients with PGP1 overexpression. The schematic diagram illustrating the reversal of MDR by anlotinib was showed in Figure 7.

To further investigate the mechanism underlying the reversal of PGP1-mediated MDR, we analyzed the effect of anlotinib on PGP1-mediated drug transport, PGP1 expression and PGP1 localization *in vitro.* Our data suggested that anlotinib inhibited the efflux of PGP1 substrates from the cells, thereby increasing the intracellular accumulation of Rho-123 and DOX. Since drug efflux depends on the energy released from ATP hydrolysis by the involved ATPase, we assessed the effect of anlotinib on PGP1 ATPase activity. Anlotinib stimulated the ATPase activity of PGP1 at a low concentration, supporting the idea that the structure of anlotinib may be similar to that of PGP1 substrates and that anlotinib could competitively inhibit PGP1 transporter activity. Furthermore, anlotinib did not affect either the expression or the localization of PGP1. These observations suggest that anlotinib activates the ATPase activity of PGP1, leading to inhibition of PGP1 efflux pump function by directly modulating the ATPase activity of the transporter, thereby reversing drug resistance. These findings are consistent with reports of other TKIs.

In conclusion, our results show that anlotinib reverses PGP1-mediated MDR by directly inhibiting PGP1 function, thus resulting in elevated intracellular concentrations of substrate chemotherapeutic drugs. Our analysis of the reversal effect of anlotinib *in vitro* and *in vivo* indicates that anlotinib may be adopted as a novel chemosensitizer to overcome MDR in patients with osteosarcoma or other types of tumors that overexpress PGP1.

## Data Availability

The original contributions presented in the study are included in the article/Supplementary Material, further inquiries can be directed to the corresponding authors.
